# Comparative Pharmacokinetics and Tissue Distribution of M10 and Its Metabolite Myricetin in Normal and Dextran-Sodium-Sulfate-Induced Colitis Mice

**DOI:** 10.3390/molecules27238140

**Published:** 2022-11-23

**Authors:** Jianchun Zhao, Wenmin Yuan, Shixiao Wang, Hongwei Zhang, Dan Chen, Xiaochen Niu, Xiaochun Liu, Li Liu, Jiangming Gao

**Affiliations:** 1 School of Medicine and Pharmacy, Ocean University of China, Qingdao 266003, China; 2 Marine Biomedical Research Institute of Qingdao, Qingdao 266073, China

**Keywords:** M10, myricetin, pharmacokinetics, tissue distribution, ulcerative colitis

## Abstract

M10, a novel myricetin derivative, is an anti-inflammatory agent designed for treatment of colitis. Here, we aim to investigate its pharmacokinetic behavior and tissue distribution in a mouse model with colitis. Pharmacokinetics and tissue distribution of M10 and its metabolite myricetin were compared in normal mice and in dextran-sodium-sulfate (DSS)-induced colitis mice. The role of fecal microbiota was also analyzed during metabolism of M10 in vitro. After oral administration, M10 was very low in the plasma of both normal and diseased mice. However, both M10 and myricetin were mainly distributed in the gastrointestinal tract, including the stomach, colon and small intestine, in physiological and pathological conditions. Significantly, M10 and myricetin were found in higher levels in gastrointestinal tracts with inflamed tissues than in normal tissues of mice. An in vitro assay revealed that 80% of M10 was metabolized to myricetin via fecal microbiota. After oral administration, M10 was not absorbed into circulation but mainly distributed in the inflamed submucosal tissues of colitic mice, where it was metabolized into myricetin to prevent colitis development.

## 1. Introduction

Inflammatory bowel disease (IBD), including ulcerative colitis (UC) and Crohn’s disease (CD), is a complex, chronic relapsing disease characterized by symptoms such as diarrhea, bloody stools, abdominal pain and weight loss [1]. Current therapeutic drugs for treatment of colitis, such as corticosteroids, immunomodulators, antibiotics and amino salicylates, have frequently failed to produce results due to disease complications [2,3,4]. Nowadays, there is an urgent need to develop new drugs with low toxicity and high efficiency in order to prevent colonic chronic inflammation and further development of colorectal cancer.

**Figure 1:**
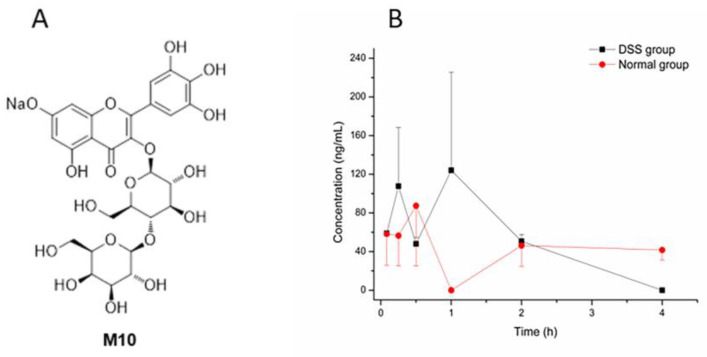
The structure of M10 and the concentration–time curve of M10. (**A**) The structure of M10. (**B**) The concentration–time curve of M10 in DSS-group mice and normal-group mice after oral administration of M10 (50 mg/kg) in plasma. Data are expressed as mean ± SD (n = 3). Plasma samples were collected at 5 min, 15 min, 30 min, 1 h, 2 h, 4 h, 6 h and 8 h after the dose.

Myricetin, a naturally occurring flavonoid, possesses multiple biological activities, such as antioxidant, anti-inflammatory, anti-carcinogenic and anti-proliferative effects [5,6,7,8]. It has been reported that myricetin inhibits intestinal tumorigenesis through inhibition of the Wnt/β-catenin pathway in adenomatous polyposis coli–multiple intestinal neoplasia (APC^Min/+^) mice [9]. Compared with myricetin, which exhibits poor water solubility (<100 ng/mL) and low bioavailability, myricetin derivative M10 (Figure 1A), designed with a hydrophilic glycosylation group, has excellent water-solubility (>0.1 g/mL), high safety (median lethal dose (LD_50_) > 5 g/kg) and high stability [10]. Recently, researchers reported that myricetin derivative M10 could inhibit intestinal tumorigenesis through inhibition of NF-kB/IL-6/STAT3 pathways [11] and also prevent chronic ulcerative colitis through inhibition of necroptosis [12]. Recently, it was reported that M10 could modify composition of gut microbiota in mice with ulcerative colitis [13].

Pathological states could change pharmacokinetic processes such as absorption, distribution, metabolism and excretion (ADME). For example, it has been reported that the pharmacokinetic parameters of chlorogenic acid were significantly changed in febrile rats; plasma concentrations and the AUC (area under the plasma concentration–time curve) especially were significantly higher than that of normal rats [14]. In clinical trials, subjects with ulcerative colitis or Crohn’s disease had 21% higher upadacitinib steady-state AUC compared with healthy subjects [15]. It has been suggested that pathologic states might influence pharmacokinetics of drugs. Therefore, uncovering pharmacokinetic data in pathological states will play an important role in clinical applications of relevant drugs and their safety and efficacy.

Recently, pharmacokinetic studies of M10 in rats were reported [16]. However, pharmacokinetic behavior and tissue distribution of M10 had not been investigated in a mouse model with colitis. It was therefore essential to clarify the characteristic of pharmacokinetics of M10 in a mouse model with colitis.

## 2. Results

### 2.1. Pharmacokinetic Study

The DSS-induced colitis mice model was successfully established through confirmation of the disease activity index (DAI) score and colon histological observation in hematoxylin and eosin (HE) staining. Then, the pharmacokinetics study was conducted. Pharmacological indicators are shown in Figures S1 and S2. Mean plasma concentration–time profiles of M10 in colitis and normal mice after oral administration of M10 (50 mg/kg) are illustrated in Figure 1B. Pharmacokinetic parameters for M10 in plasma are shown in Table 1.

C_max_: observed maximum plasma concentration; T_max_: time to observed maximum plasma concentration; AUC_0–t_: plasma exposure (area under the plasma concentration–time curve from time 0 to time of last measurable concentration); T_1/2_: half-life; MRT: mean residence time; CL: clearance.

Compared with reported pharmacokinetic studies (oral administration of M10 in rats at a dose of 100 mg/kg) of M10 (C_max_, T_max_, T_1/2_, MRT and AUC_0–t_ were 129.9 ± 52.7 ng/mL, 0.2 ± 0.1 h, 1.8 ± 0.8 h, 1.1 ± 0.4 h and 155.6 ± 147.3 ug/L * h, respectively) [16], we observed similar absorption and elimination into the blood after oral administration of M10 in our study. However, C_max_, T_max_, T_1/2_, MRT and AUC_0–t_ in DSS-group mice were different compared with those of normal-group mice. MRT in DSS-group mice was significantly different from that in normal-group mice. These results indicated that metabolic rate and elimination rate (T_1/2_ and MRT) of M10 were faster in plasma in DSS-group mice, and C_max_ was obviously more enhanced in DSS-group mice than in the normal group, which might be attributed to elevation of intestinal permeation in DSS-induced colitis mice. Furthermore, myricetin, a metabolite of M10, was also determined in plasma samples in DSS-group mice and normal-group mice, but no signals of myricetin were found in all plasma samples.

### 2.2. Tissue Distribution

**Figure 2:**
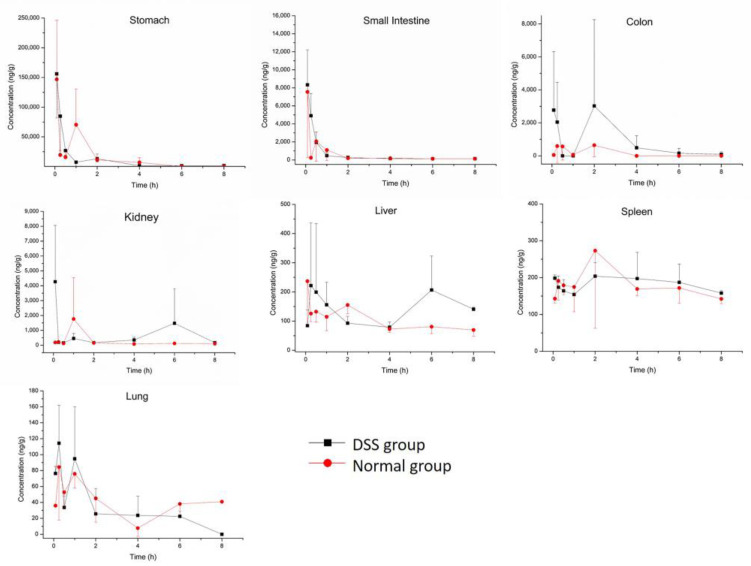
Tissue distribution profiles of M10 in DSS-group mice and normal-group mice. Data are expressed as mean ± SD (n = 3). The mice were orally administered 50 mg/kg of M10 and terminated after 5 min, 15 min, 30 min, 1 h, 2 h, 4 h, 6 h and 8 h in order to collect tissues.

**Figure 3:**
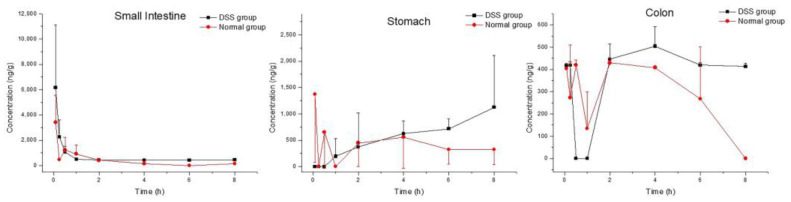
Tissue distribution profiles of myricetin in DSS-group mice and normal-group mice. Data are expressed as mean ± SD (n = 3). The mice were orally administered 50 mg/kg M10 and terminated after 5 min, 15 min, 30 min, 1 h, 2 h, 4 h, 6 h and 8 h in order to collect tissues.

Concentration and accumulation of M10 were determined in multiple tissues (heart, liver, spleen, lung, kidney, small intestine, colon and stomach) within 8 h after oral administration of M10. Myricetin was found in the colon, stomach and small intestine. The results of tissue-distribution profiles of M10 and myricetin in DSS-group mice and normal-group mice are shown in Figure 2 and Figure 3, respectively. AUC_0-t_ of M10 in DSS-group mice was observed in descending order as follows: stomach > colon > kidney > small intestine > spleen > liver > lung > heart. AUC_0-t_ of M10 in normal-group mice was observed in descending order as follows: stomach > small intestine > kidney > spleen > colon > liver > lung > heart. No signals of M10 were detected in heart samples in either DSS-group mice or normal-group mice. These results revealed that M10 was widely and rapidly distributed in the gastrointestinal tract (such as the stomach, small intestine and colon) after oral administration of M10, whether in DSS-group mice or normal-group mice. C_max_ of M10 in the small intestine, colon, kidney and liver significantly increased in DSS-group mice compared with that in normal-group mice (Table 2 and Table 3). C_max_ of M10 in the stomach, spleen, lung and heart in DSS-group mice was similar to that of normal-group mice. These results also showed that absorption of M10 in the small intestine, colon, liver and kidney was higher in DSS-group mice than that in normal-group mice. However, the liver, spleen, lung and heart were found to be poorly distributed tissues for M10 in colitis rats. It was reported that M10 significantly prevented ulcerative colitis and colorectal tumors in mice through attenuation of robust endoplasmic reticulum stress [11]. Therefore, the intestinal tract was the main target organ of M10 in treatment of ulcerative colitis and colorectal tumors in mice. We speculated that M10 probably takes local effect to relieve inflammation and diarrhea in colitis mice.

**Figure 4:**
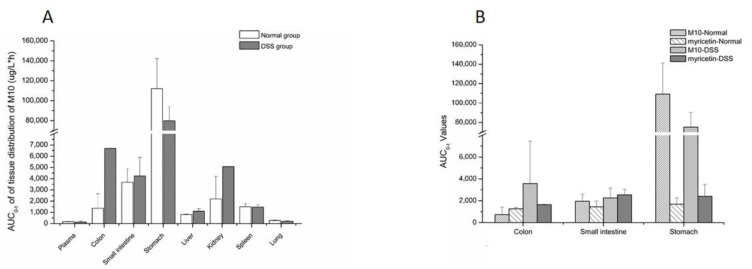
AUC_0-t_ values of M10 and myricetin in tissues. (**A**) AUC_0–t_ values of M10 in tissues after oral administration of M10 (50 mg/kg) in colitis and normal mice; (**B**) the comparison of AUC_0-t_ values of M10 and myricetin in tissues after oral administration of M10 (50 mg/kg) in colitis and normal mice.

As a presumed metabolite left by removal of lactose in M10, myricetin was detected in all tested tissues. Myricetin was found the in colon, stomach and small intestine in both the DSS group and the normal group (Figure 3 and Table 4). The maximum concentrations of myricetin in the colon (540.55 ± 45.01 ng/mL) and the small intestine (6165.64 ± 4951.21 ng/mL) were higher in DSS-group mice than in normal-group mice (438.37 ± 20.56 ng/mL and 3412.09 ± 2154.23 ng/mL, respectively). C_max_ of myricetin in the stomach (1345.17 ± 797.72 ng/mL) in the DSS group was similar to that of normal-group mice (1541.88 ± 1023.32 ng/mL). T_max_ of myriciten was delayed in the colon (3.33 ± 1.16 h) and stomach (6.00 ± 2.00 h) in the DSS group as compared with that in the normal group (colon: 1.50 ± 0.87 h, stomach: 2.06 ± 3.42 h). As shown in Figure 4B, systemic exposure of myricetin in colitis status were higher than that in normal mice. Whether in normal-group mice or DSS-group mice, AUC_0–t_ values of M10 and myricetin were comparable in the colon and small intestine, but not in the stomach, where M10 was significantly higher than myricetin.

### 2.3. Fecal Flora Participation in the Metabolism of M10

**Figure 5:**
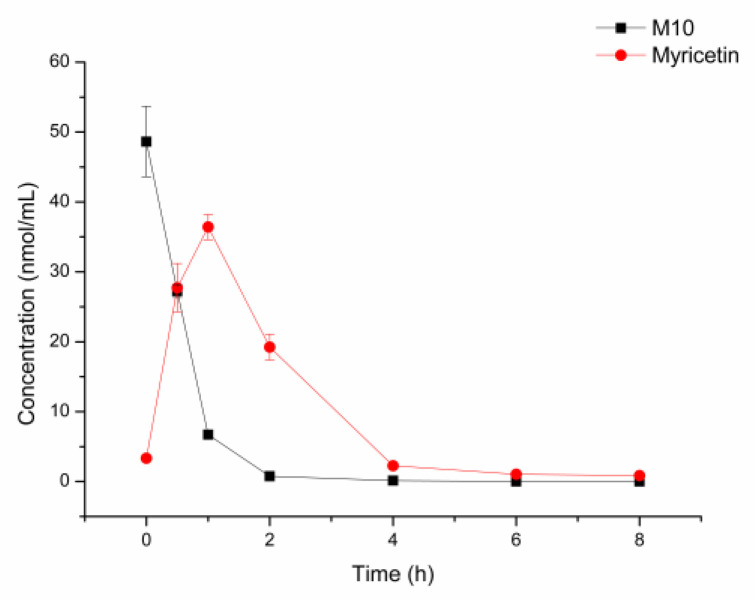
Incubation of M10 with fecal flora of normal mice at 37 °C (n = 3).

As shown in Figure 5, about 50% of M10 was metabolized by fecal flora at 0.5 h. Almost 90% of M10 was metabolized at 1 h. M10 was metabolized completely at 8 h. Meanwhile, myricetin was generated and achieved at maximal concentration at 0.5 h. The maximal concentration of myricetin was 36 nmol/mL, which is equal to 80% of the initial equivalent of M10. This revealed that M10 was metabolized by the fecal flora of the mice and most of the M10 was metabolized to myricetin in fecal flora.

## 3. Discussion

In this study, we referred to the sample-preparation method reported by Dr. Meng’s group for quantification of M10 and myricetin in mice [16]. Though M10 and myricetin feature the same precursor ion and product ion, the retention times of M10 and myricetin were 1.51 and 1.77 min, respectively. In previous pharmacodynamics studies of M10 [12] as well as in our pharmacodynamics study (Figure S3), the group with M10 at 50 mg/kg presented better activity than did other groups (such as 25 and 100 mg/kg). Due to limitation of the total amount of M10, we finally chose the dose of 50 mg/kg, which had the best activity to evaluate pharmacokinetics of M10 in mice. However, a potential limitation is that dose linearity from 25 to 100 mg/kg was unknown. According to profiles of pharmacokinetics, T_1/2_ and MRT of M10 in DSS-group mice were lower than those in normal-group mice. In colitis status, C_max_ of M10 was obviously higher than in normal mice; meanwhile, the AUC was not enhanced in colitis mice. This phenomenon might be the result of a corresponding pathologic change in colitis mice, which had higher colonic permeability than normal mice. The volume of distribution (V) of M10 in the DSS group and normal group were 142.16 ± 17.18 L/kg and 354.79 ± 78.15 L/kg, respectively, indicating that M10 was distributed into a wide range of organs and tissues. 

In the previous literature [16], M10 was shown to be rapidly absorbed in vivo, then quickly reaching C_max_ and slowly eliminated in rats. However, the pharmacokinetics of M10 in mice are different. C_max_s existed in both the DSS group and normal group, though that of the DSS group was more significant. There are several reasons that could explain this phenomenon: (1) Species differences may affect absorption and elimination between rats and mice; (2) M10 might be absorbed at different rates in different parts of the gastrointestinal tract, potentially evidenced by large amounts of M10 found in the stomach, small intestine and colon in the tissue-distribution study; (3) M10 might arrive in the small intestine of mice twice, causing it to enter the blood twice and lead to double peaks; (4) Due to increased intestinal permeability, C_max_s and T_max_s of M10 in colitis mice are higher and earlier than normal mice upon visual inspection.

Tissue-distribution studies showed that M10 was widely distributed in the tested tissues, except for the heart. Compared with normal-group mice, the AUC (Figure 4A) and C_max_ of M10 in the colon significantly increased in colitis mice, which would be beneficial to treatment of colitis. The AUC and C_max_ of M10 in the small intestine were also observed to increase in DSS-group mice as compared with normal-group mice. These results revealed that absorption of M10 in the intestinal region ascended in colitis status due to increased colonic permeability. Systemic exposure and C_max_ of M10 in main metabolic and excretory organs, such as the liver and kidney, were also enhanced in colitis status. Meanwhile, T_max_ and T_1/2_ of M10 in tissues in DSS-group mice were more delayed than those in normal mice, which indicated that metabolic rate and elimination rate of M10 in colitis status were decreased. These results were not consistent with those recorded for plasma. The main reason for this is that related pathologic changes in colitis status altered absorption and distribution of drugs in the body. Metabolic enzymes in inflammatory-bowel-disease patients were reported as changed [17] both in the liver and the intestines [18]: for example, P450 enzymes [19,20], transporters [21] and conjugative enzymes [22]. Variation of metabolic enzymes or transporters might be one of the main reasons for change of elimination rate of M10 in colitis status.

In vivo, myricetin was a typical metabolite of M10 after removal of lactose, which further metabolized until it was eliminated. The tissue-distribution results displayed that the AUC of myricetin and M10 featured the same order of magnitude (Figure 4B). The tissue distribution of myricetin in the colon, stomach and small intestine revealed that systemic exposure of myricetin in colitis status was higher than that in normal mice. 

Tissue–plasma partition coefficients (Kp) were calculated using AUC_0-t_ tissue/AUC_0-t_ plasma [23,24]. The Kp value of the colon in the DSS group was about seven times larger than that in the normal group (Table 5). However, the Kp values in the stomach were found to be 693.39 ± 343.94 and 665.54 ± 210.34 in the DSS group and the normal group, respectively. The high Kp values in the stomach indicated that M10 exhibited centralized distribution in the stomach, which suggests that M10 might have pharmacological potential for treatment of gastric inflammation in the future.

The comparative descriptions of pharmacokinetics and tissue distribution of M10 in colitis mice and normal mice have contributed to clarification of the process of M10 in vivo. First of all, the M10 prototype has low concentration in plasma. Metabolite myricetin was found in the colon, small intestine and stomach, both in normal mice and in colitis mice. Second, exposure of M10 and myricetin was enhanced in colitis mice, which might be due to increased colonic permeability. The reduced metabolic rate of M10 might be related to decreased metabolic enzymes in colitis-status mice. Third, the colon and intestine gathered large numbers of M10 and myricetin, especially in colitis mice, which would be beneficial for treatment of enteritis. These results provided useful information for further preclinical study of M10.

DSS-induced colitis mice imitated human IBD—exhibiting diarrhea, bloody feces and colonic shortening [25,26]—which was used to evaluate pharmacological activity of M10 and myricetin. Oral administration of M10 at 25, 50 and 100 mg/kg exerted a chemo-preventative effect in colitis mice (Figure S3). M10 possessed higher efficacy than myricetin and positive control mesalazine in prevention of DSS-induced colitis. Myricetin also exhibited anti-inflammatory potential in colitis mice. These results showed that both M10 and its internal metabolite myricetin have pharmacological potential for treatment of intestinal inflammatory diseases such as IBD. Using a combination of tissue distribution and pharmacodynamics, we speculated that M10 and its metabolite myricetin probably exert synergistic action on treatment of colitis.

The results of M10 in fecal flora revealed that M10 was about 80% of the mole equivalent metabolized to myricetin in fecal flora, and myricetin was proven to be the major metabolite in fecal flora in vitro. The intestine might be the main metabolic organ that gathers M10, and fecal flora played an important role in metabolizing M10.

## 4. Materials and Methods

### 4.1. Chemicals and Materials

M10 (purity ≥ 98%) was synthesized according to the reported method [10]. Myricetin (purity ≥ 98%) and azoxymethane were purchased from Sigma-Aldrich (St. Louis, MO, USA).

HPLC-grade acetonitrile and HPLC-grade methanol were purchased from Shanghai Titan Scientific Co., Ltd. (Shanghai, China). HPLC-grade formic acid (FA) was purchased from Fisher, USA. Kaempferol (internal standard, IS) was obtained from Shanghai Aladdin Biochemical Technology Co., Ltd. (Shanghai, China). DSS was purchased from MP Biomedicals, LLC. L-ascorbic acid was purchased from Sinopharm Chemical Reagent Co., Ltd. (Shanghai, China). PBS was obtained from Beijing Labgic Technology Co., Ltd. (Beijing, China). Saline (Batch No. SD20042511) was purchased from Shandong Hualu Pharmaceutical Co., Ltd. (Liaocheng, China). Deionized water was produced by a Milli-Q Reagent Water System (Millipore, Bedford, MA, USA).

### 4.2. Animal Experiment

One hundred healthy adult male C57BL/6 mice (20 ± 2 g) were provided by Beijing Weishang Lide Biotechnology Co., Ltd. (Beijing, China; license: SCXK (Jing) 2016-0009) and housed in a constant temperature of 22 ± 2 °C and a humidity environment of 55 ± 5% under a 12 h light–dark cycle, with a free diet, for 3 days. These animal experiments were approved by the Animal Ethics Committee of Ocean University of China.

### 4.3. Pharmacokinetics and Tissue Distribution of M10

All mice were assigned randomly and equally into the DSS-induced colitis group and the normal group. The DSS-induced colitis group was given free access to 2% DSS for 7 days to induce acute colitis. Normal-group mice were given saline. A total of 24 normal mice and 24 colitis mice were randomly selected and orally administrated 50 mg/kg M10 after an overnight fast. Blood samples were collected through the eyeball method at 5 min, 15 min, 30 min, 1 h, 2 h, 4 h, 6 h and 8 h after the dose and centrifuged at 8000 rpm/min for 10 min at 4 °C to obtain plasma. After withdrawal of blood, the mice were terminated, and the heart, liver, spleen, lung, kidney, stomach, colon and small intestine were collected immediately. These tissues were removed, placed on ice and washed with ice saline 3 times. Tissue moisture was absorbed by filter paper. Plasma and processed tissues were stored at −80 °C until analysis. Samples of plasma or tissue homogenate were analyzed within two weeks.

### 4.4. Biological Sample Pretreatment

In the pharmacokinetic study, the plasma samples (100 μL) from each time point of normal rats and colitis rats were added to 200 μL of pH 4.0 phosphate-buffered saline (PBS) and 10 μL of 5% L-ascorbic acid, then vortexed for 1 min before addition of 200 μL of acetonitrile containing IS (kaempferol, 10 ng/mL). Then, samples were vortex-mixed for an additional 2 min and centrifuged at a speed of 14,000 rpm/min for 10 min at 4 °C to remove plasma proteins. Finally, 2 μL of the supernatant was used for analysis.

### 4.5. In Vitro Incubation of M10 with Fecal Flora of Mice

Fresh fecal contents of mice were collected in sterile centrifugal tubes after the mice were killed. The feces were added to 30 times their weight in anaerobic broth, and the mixture was homogenized. M10 was dissolved in distilled water; then 5 μL of the M10 solution was added to 0.5 mL of the homogenate and incubated anaerobically at 37 °C in an anaerobic atmosphere generated via filling with argon. After incubation for 0 h, 0.5 h, 1 h, 2 h, 4 h, 6 h and 8 h, the reaction mixture was added to 200 μL of pH 4.0 PBS, 10 μL of 5% L-ascorbic acid and 200 μL of acetonitrile containing IS. Then, samples were vortex-mixed for 2 min and centrifuged at a speed of 14,000 rpm/min for 10 min at 4 °C. Finally, 2 μL of the supernatant was used for analysis.

### 4.6. Instrumentation and Analytical Conditions

An Agilent Technologies 1290 LC equipped with Zorbax Eclipse Plus C18 columns (2.0 × 50 mm, 5 μm) was utilized for chromatographic separation of M10 and its metabolite myricetin. The mobile phase consisted of water (A) and acetonitrile (B), both containing 0.1% formic acid, at a flow rate of 0.4 mL/min. The overrun time was 3.5 min, and gradient elution was adopted as follows: 0–0.5 min, 10% B; 0.5–2.0 min, 10–90% B; 2.0–2.5 min, 90% B; 2.5–2.51 min, 90–10% B; and 2.51–3.2 min, 10% B. The volume of injection was 2 μL, the sample temperature was maintained at 8 °C and the column temperature was maintained at 30 °C. The detection was performed with an Agilent Technologies 6460-triple quadrupole mass spectrometer with electrospray ionization (ESI). The mass spectrometer detector was set for multiple reactive monitoring (MRM) in positive mode. The correlative parameters were as follows: temperature, 350 °C; nitrogen drying gas flow, 11 L/min; nebulizer pressure, 30 psi; and capillary voltage, 4000 V. Analytes were detected at m/z 319.0/153.1, 319.0/153.1 and 287.0/153.1, with collision energy of 41 V, 41 V and 37 V for M10, myricetin and IS, respectively.

### 4.7. Data Analysis

Pharmacokinetic parameters were calculated by DAS 3.0 software (BioGuider Co., Shanghai, China). Results are presented as mean ± standard deviation (SD). Student’s *t*-test was adopted to compare two different groups at the same phase; *p* < 0.05 was considered statistically significant. Microsoft Office Excel and Origin 8.0 were used to calculate data and draw graphs. The main pharmacokinetic parameters were all calculated by non-compartmental methods.

## 5. Conclusions

In summary, the present study provided comparative pharmacokinetics and tissue distribution of M10 and its active metabolite myricetin in normal mice and DSS-induced colitis mice. These findings supply significant information about the ADME process for M10 and prospect to be conductive to guiding clinical application of M10 in colitis treatment, especially for clinical application of similar drugs in pathological states.

## Figures and Tables

**Table 1:** Pharmacokinetic parameters of M10 in DSS-group mice and normal-group mice after oral administration of M10 at a dose of 50 mg/kg (mean ± SD, n = 3).

Pharmacokinetic Parameters	DSS Group	Normal Group
C_max_ (ng/mL)	174.40 ± 55.99	115.07 ± 37.14
T_max_ (h)	0.50 ± 0.43	0.28 ± 0.21
T_1/2_ (h)	1.05 ± 0.86	1.77 ± 0.49
AUC_0–t_ (ug/L * h)	142.87 ± 83.81	170.50 ± 15.10
AUC_0–∞_ (ug/L * h)	215.81 ± 180.59	213.98 ± 22.43
V (L/kg)	142.16 ± 17.18 **	354.79 ± 78.15
CL (L/h/kg)	136.30 ± 79.38	62.57 ± 34.56
MRT (h)	0.81 ± 0.27 **	1.94 ± 0.27

** *p* < 0.01 for DSS-group mice compared with normal-group mice.

**Table 2:** Pharmacokinetic parameters of M10 in DSS-group mice tissues after oral administration of M10 at a dose of 50 mg/kg (mean ± SD, n = 3).

Parameters	Colon	Small Intestine	Stomach	Liver	Spleen	Lung	Kidney
C_max_ (ng/mL)	5742.32 ± 3986.73	8332.84 ± 3882.24	156,014.34 ± 74,478.59	417.13 ± 91.93	242.35 ± 34.51	133.65 ± 41.13	5607.33 ± 1791.25
T_max_ (h)	2.03 ± 1.96	0.08 ± 0.00	0.08 ± 0	2.25 ± 3.25	2.67 ± 1.56	0.5 ± 0.43	2.06 ± 3.42
T_1/2_ (h)	0.52 ± 0.53	7.20 ± 7.34	2.06 ± 0.70	28.14 ± 0	7.15 ± 2.17	1.13 ± 0.80 *	2.75 ± 2.05
MRT (h)	0.524 ± 0.53 *	1.45 ± 0.35	1.26 ± 0.25	4.20 ± 0.45	3.97 ± 0.20	1.88 ± 0.70	3.73 ± 1.73
AUC_0–t_ (ug/L * h)	6694.89 ± 7279.721	4248.33 ± 1659.42	79,911.87 ± 14,176.32	1099.64 ± 212.87	1470.70 ± 194.90	210.52 ± 35.58	5066.59 ± 3652.48
AUC_0–∞_ (ug/L * h)	6809.75 ± 7478.41	4989.31 ± 1007.26	83,897.97 ± 16,033.17	2163.86 ± 1645.71	2957.86 ± 594.32	233.66 ± 48.96	10,496.80 ± 11,408.11

* *p* < 0.05 for DSS-group mice compared with normal-group mice.

**Table 3:** Pharmacokinetic parameters of M10 in normal-group mouse tissues after oral administration of M10 at a dose of 50 mg/kg (mean ± SD, n = 3).

Parameters	Colon	Small Intestine	Stomach	Liver	Spleen	Lung	Kidney
C_max_ (ng/mL)	1151.71 ± 866.95	7552.80 ± 7298.53	157,843.55 ± 93,019.05	255.77 ± 129.70	337.78 ± 154.48	107.61 ± 48.38	1815.93 ± 2739.13
T_max_ (h)	0.28 ± 0.21	0.08 ± 0.00	0.39 ± 0.529	0.86 ± 1.01	1.08 ± 0.88	0.75 ± 0.43	0.44 ± 0.49
T_1/2_ (h)	0.30 ± 0.03	5.49 ± 1.39	1.01 ± 0.49	6.56 ± 4.79	7.26 ± 5.85	5.77 ± 2.12	8.48 ± 3.42
MRT (h)	1.57 ± 0.35	1.81 ± 0.65	1.45 ± 0.66	3.35 ± 0.39	3.76 ± 0.34	2.90 ± 0.98	2.97 ± 1.27
AUC_0–t_ (ug/L * h)	1363.56 ± 1297.57	3671.18 ± 1222.51	112,062.27 ± 30,214.89	801.47 ± 52.49	1489.83 ± 284.93	271.01 ± 44.61	2190.79 ± 2018.52
AUC_0–∞_ (ug/L * h)	1363.56 ± 1297.57	4183.90 ± 1192.63	112,300.22 ± 3399.32	1540.72 ± 814.39	5944.71 ± 5349.06	512.52 ± 303.74	3414.11 ± 1398.69

**Table 4:** Pharmacokinetic parameters of myricetin in DSS-group mice and normal-group mice after oral administration of M10 at a dose of 50 mg/kg (mean ± SD, n = 3).

Tissue	C_max_		T_max_		AUC_0–t_	
	DSS Group	Normal Group	DSS Group	Normal Group	DSS Group	Normal Group
Colon	540.55 ± 45.01 *	438.37 ± 20.56	3.33 ± 1.16	1.50 ± 0.87	3066.05 ± 84.60 **	2362.76 ± 244.96
Stomach	1345.17 ± 797.72	1541.88 ± 1023.32	6.00 ± 2.00	2.06 ± 3.42	4510.22 ± 2033.43	3168.06 ± 1056.16
Small Intestine	6165.64 ± 4951.21	3412.09 ± 2154.23	0.08 ± 0.00	0.08 ± 0.00	4764.72 ± 960.37 *	2701.90 ± 999.18

*p < 0.05, **p < 0.01 compared with normal-group mice.

**Table 5:** Tissue–plasma partition coefficients (Kp) of M10 after oral administration of M10 at a dose of 50 mg/kg in the normal and DSS groups (mean ± SD, n = 3).

Group	Colon	Small Intestine	Stomach	Liver	Kidney	Spleen	Lung
DSS group	57.07 ± 64.86	31.89 ± 5.98	693.39 ± 343.94	9.29 ± 4.94	42.41 ± 34.06	12.92 ± 6.74	1.91 ± 1.25
Normal group	7.90 ± 7.06	22.07 ± 9.47	665.54 ± 210.34	4.71 ± 0.16	13.66 ± 13.74	8.72 ± 1.30	1.61 ± 0.43

## Data Availability

The data presented in this study are available on request from the corresponding authors.

## Supplementary Materials

The following are available online at https://www.mdpi.com/article/10.3390/molecules27238140/s1, Figure S1: DAI score of evaluation of enteritis models; Figure S2: Colon histological observation in HE staining; Figure S3: Pharmacodynamics of M10 and myricetin; Table S1: Linear equation, correlation coefficients (R^2^), linear ranges and lower limit of quantification (LLOQ) of M10 and myricetin in biological samples of mice.

## Sample Availability

Samples of the compounds are not available from the authors.
