# Supplementary material for: Comparative Pharmacokinetics and Tissue Distribution of M10 and Its Metabolite Myricetin in Normal and Dextran-Sodium-Sulfate-Induced Colitis Mice
Source: Molecules. 2022 Nov 23;27(23):8140. doi: 10.3390/molecules27238140 (PMC9740244; doi:10.3390/molecules27238140)
Supplement: Supplementary file 1 [file molecules-27-08140-s001.zip › molecules-2007131-supplementary.pdf]

Supporting Information

Comparative Pharmacokinetics and Tissue Distribution of M10 and Its Metabolite Myricetin in Normal and Dextran-Sodium-Sulfate-Induced Colitis Mice

Jianchun Zhao [1,2,*], Wenmin Yuan [2], Shixiao Wang [1], Hongwei Zhang [1,2], Dan Chen [1], Xiaochen Niu [1], Xiaochun Liu [2], Li Liu [2] and Jiangming Gao [2]

[1] School of Medicine and Pharmacy, Ocean University of China, Qingdao 266003, China
[2] Marine Biomedical Research Institute of Qingdao, Qingdao 266073, China
* Correspondence: zhaojianchun@ouc.edu.cn

1. The evaluation results of colitis mice model establishment

The DSS-induced colitis mice model was successfully established by confirmation of disease activity index (DAI) score and the colon histological observation in HE staining. Then the pharmacokinetics study was conducted. These pharmacological indicators were showed in Figure S1 and Figure S2.

As shown in Figure S1, compared with the normal rats, the DAI score were significantly increased in the DSS-induced enteritis mice.

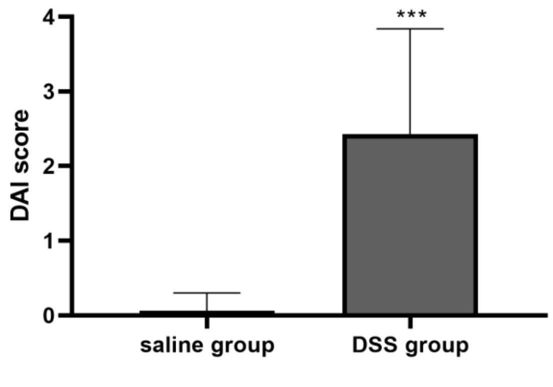

Figure S1 The DAI score of evaluation of enteritis models. The data were presented as mean ± SD (n = 10).

The mice were killed on the 7th day and the colon tissue was separated to perform HE staining for pathological analysis. As showed in Figure S2, the histological studies showed that in the normal group mice, the structure of intestinal mucosa was clear, and the structure of mucosa, submucosa, muscle layer and serosa was normal, the cell

morphology was good, and there was no erosion and ulcer formation. In the DSS group mice, the normal structure of the intestinal mucosa disappeared, and the epithelial cells of the mucosa degenerated, necrotized and shed. A large number of inflammatory cells infiltrated into the mucosa and submucosa, and the muscular layer and serosa were still intact. Those data could prove on the successful establishment of colitis models.

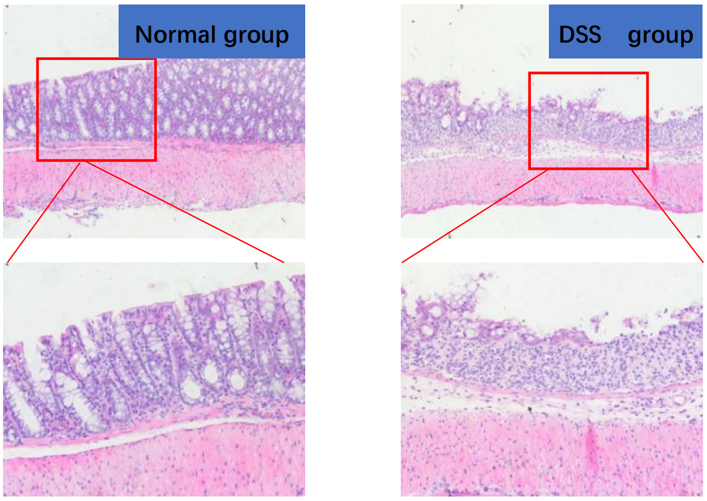

Figure S2 Colon histological observation in HE staining.

2. The results of calibration curve

Table S1. The linear equation, correlation coefficients (R^2), linear ranges and lower limit of quantification (LLOQ) of M10 and myricetin in mice biological samples

Sample	Calibration curve	R^2	Linear range (ng/mL)	LLOQ (ng/mL)
M10				
Plasma	Y=0.037729X-0.057078	0.9999	50-2000	50
Colon	Y=0.075009X+0.920451	0.9964	10-2000	10
Small intestine	Y=0.078161X+0.415449	0.9979	10-2000	10
Stomach	Y=0.300744X-0.012392	0.9999	20-2000	20
Heart	Y=0.199407X+0.896700	0.9971	20-2000	20
Liver	Y=0.555727X-0.872651	0.9985	10-2000	10
Lung	Y=0.400216X+0.209389	0.9997	10-2000	10
Kidney	Y=0.430102X-1.172678	0.9983	50-2000	50
Spleen	Y=0.348070X-1.333610	0.9997	50-2000	50

myricetin				
Colon	Y=1.189365X-15.849125	0.9958	10-2000	10
Small intestine	Y=1.189365X-15.849125	0.9958	10-2000	10
Stomach	Y=1.189365X-15.849125	0.9958	10-2000	10

3. Colonic chronic inflammation model and treatment

A total of forty-two mice were randomly divided into 7 groups. Group a: normal control mice treated with drinking water; Group b: DSS-induced colitis model treated with drinking water; Group c: mice exposed to DSS treated with 100 mg/kg of mesalazine; Group d-f: mice exposed to DSS treated with 25, 50 and 100 mg/kg of M10, Group g: mice exposed to DSS treated with 100 mg/kg of myricetin; respectively. The colonic chronic inflammation model was established according to previous report [1]. Briefly, mice were received drinking water containing 1% DSS for 7 days, followed by receiving drinking water for 7 days. Then mice were accepted three additional DSS treatment cycles. The pharmacological treatments were performed by gavage once a day for consecutive 2 weeks from the second day after DSS. Mice were observed daily for any signs of illness. Mice were sacrificed after last administration. The colorectal tissues were taken out and measured, and then snap-frozen in liquid nitrogen or placed in formalin for histopathology analysis.

4. Anti-chronic colonic inflammation activity of M10 and myricetin

Anti-chronic colonic inflammation activities of M10 and myricetin were evaluated in the DSS-induced mice model. The average length of colon in normal mice was 9.2 cm, while the average length of colon in colitis mice was shorten to 7.6 cm ($p < 0.05$ vs. normal mice) (**Figure S3A**). Compared with the model group, the lengths of colon increased when the mice were given M10 at a dose of 25, 50 and 100 mg/kg, respectively. Mice treated with M10 at 50 mg/kg presented colorectal length by 8.2 cm, which indicated that M10 had higher activity than other groups. Histopathologic analysis revealed severe inflammatory lesions in colonic tissues in model mice, such as structural changes, denudation and collapse of villi, inflammatory cellular infiltration, multiple erosions, crypt abscesses and regenerating epithelium. When mice treated with

M10, the colon tissues demonstrated the integrity and normal structure (**Figure S3B**) and the characteristics were also observed in myricetin group.

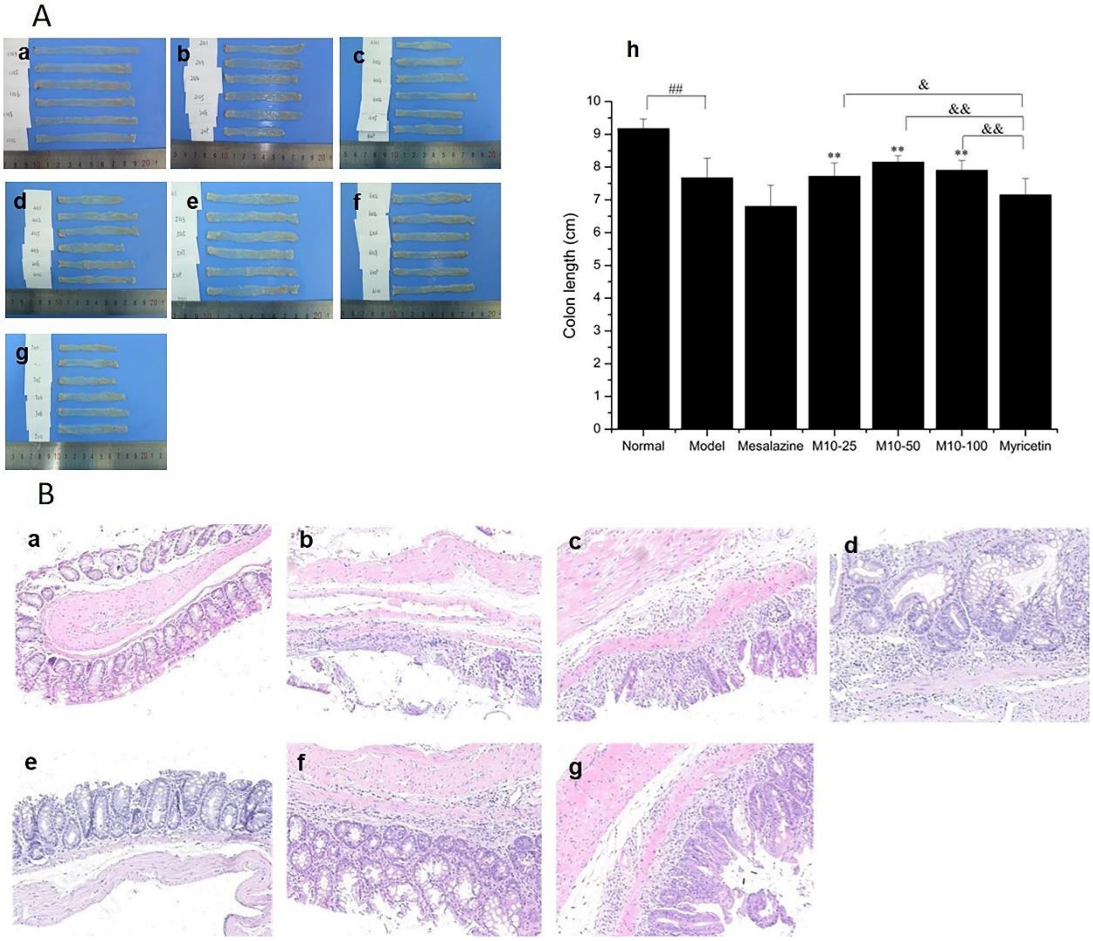

Figure S3. Pharmacodynamics of M10 and myricetin. A: Gross views of the colons and the lengths of the colons in each group at the end of administration. (a) Colons from the normal mice; (b) Colons from DSS-induced colitis model treated with drinking water; (c) Colons from mice exposed to DSS and treated with 100 mg/kg of mesalazine; (d-f) Colons from mice exposed to DSS and treated with 25, 50 and 100 mg/kg of M10, respectively; (g) Colons from mice exposed to DSS and treated with 100 mg/kg of myricetin (h) The length of colons from each group (n = 6). $^{\#\#}p < 0.01$ between normal and model mice. $^{**}p < 0.01$ between model and drug-treated mice. $^{\&}p < 0.05$, $^{\&\&}p < 0.01$ between M10 and myricetin. B: Representative of the HE-stained colonic tissues. (a) Normal

group; (b) Colitis model group; (c) Mesalazine-treated mice; (d-f) M10-treated mice by 25, 50 and 100 mg/kg; (g) Myricetin-treated mice.

References:

1. Miao, R. R.; Zhan, S.; Hu, X. T.; Yuan, W. M.; Wu, L. J.; Cui, S. X.; Qu, X. J. Myricetin and M10, a myricetin-3-O-β-d-lactose sodium salt, modify composition of gut microbiota in mice with ulcerative colitis. *Toxicol. Lett.* **2021**, *346*, 7–15.
